# Can social media be used to increase fruit and vegetable consumption? A pilot intervention study

**DOI:** 10.1177/20552076241241262

**Published:** 2024-04-24

**Authors:** Lily Hawkins, Claire Farrow, Meshach Clayton, Jason M Thomas

**Affiliations:** 1 Department of Public Health and Sports Sciences, Medical School, 3286 University of Exeter, Exeter, UK; 2 School of Psychology, College of Health and Life Sciences, 1722 Aston University, Birmingham, UK

**Keywords:** Social media, social norms, food consumption, healthy eating, fruit, vegetables

## Abstract

**Background:**

Exposure to social norms about fruit and vegetable intake has been shown to increase individuals’ consumption of these foods. Further, exposure to socially endorsed ‘healthy’ food posts can increase consumption of low energy-dense (LED), relative to high energy-dense (HED) foods. The current pilot study aimed to investigate whether exposure to healthy eating (vs. control) social media accounts can shift normative perceptions about what others eat, eating intentions and self-reported food consumption.

**Methods:**

In a 2 (condition) × 2 (type of food consumed) mixed factorial design, 52 male and female students were asked to follow either healthy eating (intervention) or interior design (control) Instagram accounts over a two-week period. Baseline and post-intervention measures assessed normative perceptions of Instagram users’ consumption of fruit and vegetables (LED foods), and energy dense snacks and sugar sweetened beverages (HED foods). Participants’ intentions to consume, and self-reported consumption of these foods, were also measured.

**Results:**

There were no significant changes in perceptions about what others eat, or participants’ own eating intentions (*p*s > 0.05). However, the intervention increased participants’ self-reported consumption of LED foods by 1.37 servings (per day) and decreased consumption of HED foods by 0.81 items (per day), compared to the control condition (*ps *< 0.05).

**Conclusions:**

This novel pilot study demonstrates that a social norm–based social media intervention can successfully encourage healthier eating, with a large effect after two weeks. Certain social media platforms may therefore provide a viable tool for nudging healthy eating. Future work will aim to replicate these findings in a larger and more diverse sample.

## Introduction

In 2018, only 28% of the UK population consumed five-a-day of fruit and vegetables, with 16–24 year olds consuming just three portions a day, on average.^1^ Low fruit and vegetable consumption has been linked to various poor health outcomes including heart disease, cancer and stroke, whereas higher fruit and vegetable consumption has been linked to improved well-being and may help to prevent weight gain.^2,3^ Thus, it is important to identify effective methods by which to promote and encourage the consumption of these foods, especially for young adults.

One way to encourage fruit and vegetable consumption is to utilise social influences, such as social norms, which have frequently been shown to be associated with, and to enhance, fruit and vegetable consumption.^4–9^ Indeed, social norms have frequently been used as a basis for interventions aiming to reduce alcohol consumption (e.g., Refs.^10–12^), but less attention has focussed on applying social norms as a basis for healthy eating interventions. Some studies have started to consider this, for instance, highly rated healthy foods were found to lower preferences for unhealthy foods, but not consumption of confectionary.^13^ Additionally, social norm posters in workplace canteens, stating that ‘most people here choose to consume vegetables with their lunch’, increased the number of meals bought with vegetables post-intervention.^14^ This demonstrates the substantial potential of this approach in real-life settings; however, the development and deployment of social norm–based interventions to encourage fruit and vegetable consumption is still largely unrealised. Further, it is not yet clear what types of social norm–based interventions might be most effective.

One way in which norms may be communicated is social media, which is now highly prevalent within many people's lives.^15^ Indeed, social media platforms may present an opportunity to deliver normative-based interventions. As an example, Sharps and colleagues^16^ considered whether a social media intervention using portion size norms could decrease portion sizes of high energy-dense (HED) snacks, through posting of ‘other peers’ snacks (and hence, the portion size of the snacks), using confederate accounts. The first of their two pilot interventions reduced participants’ own desired HED portion size; however, the second intervention had no effect on desired portion size between intervention and control participants. Although the evidence here is mixed, the approach is worthy of further investigation, particularly utilising real social media accounts (rather than fake confederates etc.) to improve the external validity of such approaches. Additionally, research to investigate the effect of normative interventions on low energy-dense (LED) food consumption is required.

Another important consideration here is the mechanism by which social norms affect eating behaviour. The Theory of Planned Behaviour^17^ (TPB) would suggest that such normative interventions may affect our perceptions of what others do, and thereby affect our intentions to consume food and our actual eating behaviour. Indeed, the TPB has been applied to healthy eating behaviour^18^ as well as healthy eating interventions with success,^19^ and intentions to consume fruit and vegetables have previously been found to be affected by social norms,^8^ but to our knowledge, this has not been formally investigated in the context of social media and so warrants further investigation.

### The present study

This intervention pilot study therefore aimed to nudge healthy eating by encouraging fruit and vegetable consumption in habitually low consumers of these foods because they are likely to receive the greatest benefit.^20^ Participants were asked to follow real healthy eating accounts (i.e., accounts in which over half of the social media posts contained photos of fruits or vegetables) or real interior design accounts (as a control), on the social media platform Instagram. These accounts were selected from a pre-approved list by the research team (see Materials section). To ensure scalability for individual participants, they were asked to add 5% of the number of accounts that they were currently following (so, if for example, a participant followed 300 accounts, they were asked to follow 15 extra healthy eating or control accounts).

#### Aims and hypotheses

The first aim of this pilot study was to investigate whether exposure to the healthy eating accounts (vs. control) would increase participants’ perceptions of the consumption of fruits and vegetables (i.e., LED foods) by other Instagram users, as well as their own intentions to consume, and self-reported consumption of these foods, across a two-week period. Secondly, we aimed to investigate whether a change in perceptions about other Instagram users’ LED consumption, due to the intervention, would in turn affect participants’ self-reported consumption of LED foods. High energy-dense foods were also examined, for both aims, as a comparator. It was hypothesised that exposure to additional healthy eating accounts would result in participants perceiving Instagram users to consume more LED foods, and for participants to intend to consume and to self-report consuming more LED foods, compared to exposure to additional control accounts. Further, it was hypothesised that the intervention would increase participants’ LED consumption as a result of an increase in normative perceptions (i.e., following intervention accounts would increase the perceptions of the amount of LED foods that Instagram users eat, and consequently increase participants’ own consumption of LED foods).

## Method

### Participants

A total of 60 undergraduate and post-graduate students (62% women; 38% men; mean age = 22.0, SD = 2.3), completed the study. Participants were recruited via Aston University's Psychology Research Participation System, social media and emails sent to students via departmental administrators. Participants were screened from taking part in the study if they did not meet the following criteria: low habitual consumers of fruit and vegetables (i.e., consume less than three portions of fruit and vegetables a day), aged 18–65 years, non-smokers, current Instagram users and check their Instagram account regularly (i.e., more than three days a week, assessed via the question ‘How many days a week do you check your Instagram account?’), and not follow more than 500 accounts (to ensure that the intervention – following additional accounts – was practicable for participants). Additionally, participants with a BMI outside of 18.5–40.0, or those who reported having eating disorders, food allergies or diabetes, were excluded from the final sample, as these participants are more likely to have atypical eating patterns. As a result, eight participants were removed, leaving a final sample of 52. Participants were awarded course credits for their participation or entered into a prize draw to win one of three £100 Amazon vouchers. Ethical approval was granted by Aston Life and Health Sciences Ethics Committee (#1512), and the study was conducted in accordance with the ethical standards of the 1975 Declaration of Helsinki, as revised in 1983. Informed consent was obtained from all participants.

### Sample size

Our power analysis was configured with α = 0.05, to detect a large effect size (*f* = 0.8), and with power set at 80%, suggesting the required sample size was 42 for ANOVA. However, based on potential issues with intervention compliance and drop-out from the study, we aimed to recruit 60 participants in total.

### Design

The study employed a 2 (Between-subjects factor ‘Condition’; intervention or control) × 2 (Within-subjects factor ‘Type of Food’; LED or HED) mixed factorial design. Condition consisted of asking participants to follow certain Instagram accounts (see Materials for details), which either contained a high proportion of highly liked posts of LED foods for those in the intervention condition or highly liked posts of interior design, to act as a control condition. The dependent variables were changes in: (a) perception of Instagram users’ consumption of LED foods (fruit and vegetables combined) and HED foods (HED snacks and SSBs combined); (b) participants’ intentions to consume LED foods and HED foods; and (c) self-reported consumption of LED and HED foods (post-intervention scores were subtracted from baseline (i.e., pre-intervention) scores to provide change scores). Additionally, a mediation model was used to examine whether a change in normative perceptions about other Instagram users’ LED consumption mediated the relationship between healthy eating intervention accounts (vs. control) and participants own LED consumption. This was carried out with condition as the predictor variable, normative perceptions of Instagram users’ consumption of LED foods as the mediating variable, and participants’ own LED consumption as the outcome variable. The same model was also applied to HED foods (i.e., perception of HED food consumption as the mediator, and participants’ HED consumption as the outcome variable).

### Materials

The intervention was conducted using the online survey platform Qualtrics and comprised a series of questionnaires presented in the order below:

*Visual Analogue Scales^9^* *(VAS):* This scale assessed baseline and post-intervention mood and appetite, using the following items: ‘alertness’; ‘drowsiness’; ‘light-headedness’; ‘anxiety’; ‘happiness’; ‘nausea’; ‘sadness’; ‘withdrawn’; ‘faint’; ‘hunger’; ‘fullness’; ‘desire to eat’ and ‘thirst’. Participants were asked to indicate on a scale from 0–100 (0 = not at all, 100 = very much) how they felt, for each item, at the present time of the study. Baseline VAS scores were considered as potential covariates.

*The Usual Food and Drink Intake Questionnaire^21^* *(UFDIQ):* Measured participants’ own habitual consumption and liking of fruit, vegetables, energy-dense snack foods and sugar sweetened beverages, using open-ended questions (e.g., ‘*How many servings of vegetables do you typically eat a day?’*) and VAS items (e.g., ‘*on a scale from 0 (Not at all) to 100 (Very much), how much do you like eating vegetables*?*’*). Baseline consumption was considered as a potential covariate.

*Lifestyle Questionnaire^9^:* This was collected only at baseline and to measure sample characteristics regarding age, gender, previous and current history of eating disorders, ethnicity, socio-economic status and family income, and to exclude participants based on study criteria (e.g., allergies).

*Height and weight:* Post-intervention, participants were asked to report their height in metres and weight in kilograms. This information was used to calculate BMI to consider this as a covariate.

*Intentions to consume* LED and HED foods were measured using two questionnaires. The first, as used by Stok and colleagues,^8^ measured intentions to consume fruit and vegetables over the following two weeks, using four items (e.g., ‘*I [intend/plan/want/expect] to eat sufficient fruit and vegetables over the coming time*’), with sufficient being defined as two portions a day by the authors. These were rated on a five-point Likert-type scale from ‘completely agree’ (5) to ‘completely disagree’ (1). A second questionnaire was adapted from the UFDIQ,^20,21^ to ask how many servings of vegetables, fruit and HED snacks, SSBs participants intended to consume per day, over the two-week period. Participants responded with an open-ended response, giving a number to indicate their response. After validating the second measure against the first, data from the second measure were analysed here (as this was the more comprehensive measure).

*Identification with Instagram users* was measured using visual analogue scales,^8^ to measure how strongly participants identify as and affiliate themselves with Instagram users. Two statements (e.g., ‘*I identify with Instagram users’; ‘I feel a strong connection to Instagram users’*) were measured on a 0 (‘Not at all’) to 100 (‘Very Much’) scale*.*

*Social Networking Use^22^:* This assessed Instagram and other social media use within the sample. This scale used nine items including frequency of use, the number of accounts ‘followed’, the number of other social media accounts participants use, and how often, using a combination of open-ended questions (e.g., ‘*Roughly how many followers do you have?’*) and response scales (e.g., ‘*How long do you typically spend on Instagram?’*, with responses measured on a five-point Likert scale).

*Three-Factor Eating Questionnaire-R21^23^* *(TFEQ-R21):* Measured uncontrolled eating (e.g., ‘*Sometimes when I start eating, I can’t seem to stop’*), cognitive restraint (e.g., ‘*I don’t eat some foods because they make me fat’*) and emotional eating (e.g., ‘*I start to eat when I feel anxious’*) as potential covariates. Participants indicated their response on a four-point Likert-type scale (‘definitely true’, ‘mostly true’, ‘mostly false’ and ‘definitely false’).

*The Student Food Attitudes Form (SFAF):* This scale uses open-ended questions to measure perceived descriptive and injunctive norms (e.g., ‘*How many servings of [vegetables] do you think a typical Instagram user [should] eat a day?)* for each food and drink^9^ (fruit, vegetables, HED snacks and sugar sweetened beverages), where participants respond with a number (e.g., ‘3’), to indicate number of servings. A Visual Analogue Scale (measured from 0, ‘Not at all’, to 100 ‘Very much’) was also used to measure perceived liking norms for each food type (e.g., ‘*How much do you think a typical Instagram user enjoys eating vegetables*?’). To measure norms about frequency of consumption, the question ‘*how often do you think a typical Instagram user eats/drinks…’* was used (as in Robinson et al.^24^). Answers were rated on a five-point scale from ‘Never’ (0) to ‘Daily or almost daily’ (4).

*Experimental Manipulation* – *Instagram accounts:* A database of 50 Instagram accounts was compiled (25 healthy food and 25 interior design accounts) by searching Instagram using hashtags (#healthyeating, #healthyrecipes, #healthyfood and #interiordesign and #interior). Accounts were excluded if posts contained people eating, promoted a specific diet, were from well-known celebrities or high-profile individuals, or included photos of kitchens or dining scenarios, to prevent these factors from biasing behaviour. The maximum number of accounts that participants could report following to take part was 500, such that adding 5% of accounts (i.e., 25 at maximum) did not become burdensome and reduce compliance. To try to represent the social media environment, and the range of accounts that participants may follow organically, accounts had a range of followers (min. = 28,400; max. = 5.1 million; mean = 1,517,207 followers). Similarly, accounts were only selected for inclusion in the database if posts typically received at least 1000 likes, hence, they were ostensibly popular and influential accounts. Participants were randomised to condition by the researcher using randomiser.org and were emailed a list of accounts by the researcher, which they were instructed to follow within 24 h.

*Demand and compliance check:* Post-intervention, participants were asked what they thought the purpose of the study was (demand check) using an open-ended response. Participants were also asked at baseline for their Instagram name, so that a researcher could check if they appeared in the list of followers for their specified accounts. At the end of the study, participants were also asked how many of the accounts they had followed from the list they had been sent, to verify how likely participants were to be exposed to the intervention. Results of the compliance check are reported in ‘Results’ section.

### Procedure

Participants were told that the study was aiming to investigate Instagram use and lifestyle habits. After completing a screening questionnaire via email, including basic demographic information and inclusion/exclusion criteria stated above, participants were invited to complete the first part of the online study through Qualtrics. Participants completed the questionnaires (above) and were then randomly assigned to a condition and provided a number of accounts to follow (from the pre-specified list by the research team) via email, with instructions to follow these accounts within 24 h, for a two-week period. After this two-week period, participants were contacted, or booked a time slot, and invited to complete the second, shorter part of the study (Visual Analogue Scales, UFDIQ, Social Networking questionnaire, intentions to consume, identification with Instagram users and SFAF), as well as report their height and weight (for BMI), demand awareness and compliance check. Participants were then fully debriefed as to the exact aims of the study, thanked for their time and either credited or entered into the prize draw to win one of the vouchers. Data was collected from April to June 2020. Each part took no longer than 15 min.

### Analysis

*Main Analyses:* Change scores were analysed with independent t-tests to examine differences between the control and intervention conditions for perceptions, intentions and self-reported consumption of LED foods (fruit and vegetables) and HED foods (HED snacks and SSBs). A planned mediation analysis using PROCESS 16.3 v2 (Hayes), with Bootstrapping at 5000, was also conducted to examine whether a change in normative perceptions about what Instagram users consume (perceived descriptive, injunctive, liking and frequency norms for food, each analysed separately) mediated the effect of condition on the change in participants own LED and HED consumption (change scores for normative perceptions were calculated and used in analysis).

*Covariates for main analyses:* Theoretical covariates including baseline consumption, baseline VAS mood and appetite, TFEQ-21R eating styles and BMI, were examined using correlations and also examined for baseline differences using ANOVA and t-tests (as appropriate), as these may all affect food consumption and choice. Variables were to be included as covariates if they correlated significantly (*p *< 0.05) with both LED and HED consumption outcome measures. As this was not the case, they were not used as covariates.

*Additional Analyses:* Independent t-tests were used to examine differences in key baseline participant characteristics across conditions as a randomisation check. For VAS data, a principal components analysis (PCA) with Varimax rotation was carried out on the VAS items (mood and appetite). This yielded four factors with eigenvalues >1, which accounted for a total of 72% of the variance. Factors included ‘Appetite’ (hunger, full (reverse coded), desire to eat), ‘Negative affect’ (sad, anxious, withdrawn, nausea, faint, happy (reverse coded)), ‘Arousal’ (alert (reverse coded), lightheaded, drowsy) and ‘Thirst’. Once factors were identified, aggregate scores for each dimension were computed, inverting scores for items where relevant. A 2 (condition) × 2 (time) mixed ANOVA was then used to examine differences for each of the VAS PCA mood and appetite scores. The same 2 × 2 ANOVA model was also applied to Instagram affiliation scores.

## Results

### Participant characteristics

The final sample comprised 52 participants (28 in the control condition and 24 in the intervention condition). Forty-two percent (*n* = 22) of the sample were Asian, 31% (*n* = 16) were from a white ethnic group, 17% (*n* = 9) were from a black ethnic group and 9% (*n* = 5) were mixed ethnicity. For income, 48% (*n* = 25) reported their total family income as between £25,000 and £40,000, 25% (*n* = 13) between £15,500 and £25,000, 15% (*n* = 8) above £40,000 and 11% (*n* = 6) below £15,500. For SES, 62% (*n* = 32) of the sample classed themselves as middle class, 29% (*n* = 15) lower-middle class and 9% (*n* = 5) lower class. Forty eight percent (*n* = 25) of the sample were consumers of alcohol. BMI and TFEQ-R21 subscale scores were compared between conditions with independent *t*-tests; there were no significant differences (data reported in Table 1).

**Table 1.** Key participant characteristics split by condition (means and standard deviations).

Measure	Control condition, M (SD)	Intervention condition, M (SD)	*p* value
BMI	24.1 (3.5)	23.8 (3.7)	0.77
TFEQ-R21 UE	2.3 (.4)	2.2 (.6)	0.40
TFEQ-R21 CR	2.3 (.5)	2.2 (.6)	0.67
TFEQ-R21 EE	2.2 (.8)	2.0 (.9)	0.29

BMI: body mass index; TFEQ-R21: Three-Factor Eating Questionnaire-R21; UE: uncontrolled eating; CR: cognitive restraint; EE: emotional eating.

### VAS appetite and mood scores

Mixed ANOVA of VAS scores showed that for VAS appetite, thirst, negative affect and arousal, there were no significant main effects of time or condition, and no significant interactions between time and condition (all *p*s > 0.05; see Table 2 for means and SD).

**Table 2.** VAS mood and appetite scores split by condition and time (means and standard deviations).

VAS factor	Control condition		Intervention condition	
	T1	T2	T1	T2
	M (SD)	M (SD)	M (SD)	M (SD)
Appetite	48.5 (5.5)	54.1 (6.1)	56.1 (5.9)	40.7 (6.6)
Thirst	63.6 (5.4)	59.2 (6.2)	62.0 (5.8)	63.4 (6.6)
Negative affect	18.4 (3.1)	18.9 (3.3)	26.8 (3.3)	19.3 (3.5)
Arousal	23.4 (4.1)	26.9 (4.7)	30.3 (4.5)	25.4 (5.1)

### Social media use

The modal time spent on social media was ‘over an hour’ per day (46% of participants), and the vast majority of participants used other social media in addition to Instagram (94% of participants). Turning to Instagram specifically, all participants had an Instagram account (100%), using it, on average, for more than three days a week (as stated in the inclusion criteria). The mean number of followers for participants’ accounts at baseline was 261 (SD = 274.4) and the mean number of accounts participants followed was 251 (SD = 230.9).

### Affiliation with Instagram users

For affiliation with Instagram users, a 2 (condition) × 2 (time) ANOVA revealed that for items relating to identification with Instagram users, there was a significant main effect of time (*F*(1) = 4.56, *p* = 0.04, η² = .08), whereby at baseline, participants reported higher average identification with Instagram users (mean = 53.4) than post-intervention (mean = 44.0). Further, there was also a significant main effect of condition (*F*(1) = 11.97, *p* = 0.001, η² = .19), whereby those in the intervention condition reported higher scores (mean = 59.0) than those in the control condition (mean = 38.4). However, there was no significant interaction between time and condition (*F*(1) = 2.28, *p* = 0.14, η² = .04).

### Compliance and demand check

When asked how many accounts they had followed from the list provided, 85% of participants responded, of which, 80% (*n* = 40) reported following the correct number of accounts given to them for the intervention. There were no significant differences in compliance between conditions (χ^2^(1) = 1.62, *p* = 0.20). While 22% (*n* = 13) of participants partly guessed that the aims of the study were to try and encourage ‘healthier eating’ using Instagram, none of the participants correctly guessed the precise aims of the study.

### Main analysis

#### Change in normative perceptions and intentions to consume

For change in normative perceptions, there were no significant differences between conditions for any of the measures (all *p*s > 0.05 – see Table 3). Similarly, there were no significant differences between conditions for change in intentions to consume LED foods (*t*(49) = 0.48, *p *= 0.64) or HED foods (*t*(48) = 1.00, *p *= 0.30; see Table 3).

**Table 3.** Change in normative perceptions of, and intentions to consume LED and HED foods, split by condition (means and standard deviations).

Measure	Condition	
	Control	Intervention
	M (SD)	M (SD)
*Normative perceptions*		
LED Descriptive norm	−0.4 (1.1)	0.2 (1.6)
LED Injunctive norm	−0.4 (1.5)	−0.4 (1.2)
LED Liking norm	4.0 (41.2)	11.4 (28.8)
LED Frequency norm	−0.2 (1.1)	−0.1 (0.7)
HED Descriptive norm	0.0 (1.0)	−0.1 (1.1)
HED Injunctive norm	0.2 (1.3)	−0.3 (1.0)
HED Liking norm	−5.3 (28.4)	−8.0 (35.0)
HED Frequency norm	0.1 (0.8)	0.0 (1.4)
*Intentions to consume*		
LED Foods	1.4 (3.1)	1.1 (1.4)
HED Foods	0.0 (4.4)	−1.0 (2.1)

#### Change in self-reported consumption

**Figure 1.**
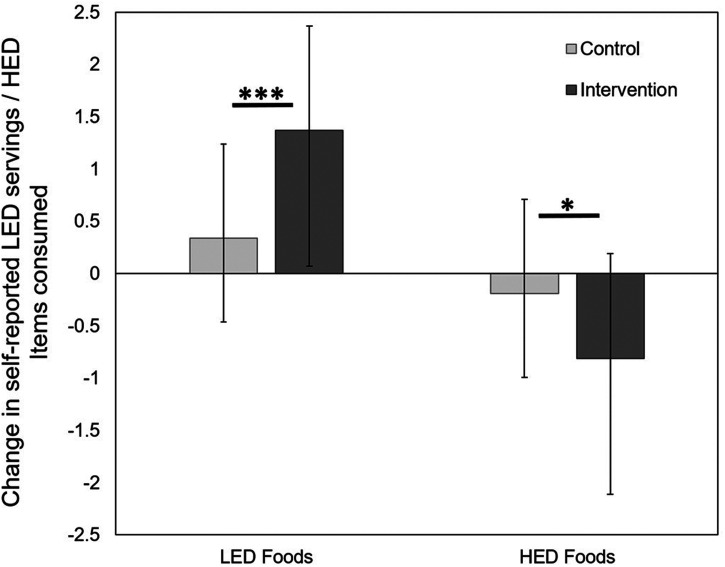
Differences in self-reported servings of LED and items of HED foods consumed over the two-week period, by those in the control and intervention conditions (error bars = SD). LED: low energy-dense; HED: high energy-dense.

T-tests revealed significant differences between conditions for self-reported consumption of LED foods (*t*(49) = −3.76, *p *< 0.001; Cohen's *d* = 1.1), whereby those in the intervention condition increased their consumption by an average of 1.37 (SD = 1.0) servings of fruit and vegetables per day across the two-week time period, compared to the those in the control condition who increased their consumption by 0.34 (SD = 0.9) servings (see Figure 1). There was also a significant difference between conditions for self-reported HED consumption (*t*(47) = 2.06, *p *= 0.045; Cohen's *d* = 0.6), whereby those in the intervention condition decreased their consumption by an average of 0.81 (SD = 1.3) items of HED snacks and sugar sweetened beverages per day across the two-week time period, compared to the those in the control condition who decreased their consumption by 0.19 (SD = 0.8) items (see Figure 1).

#### Mediation analysis

**Figure 2.**
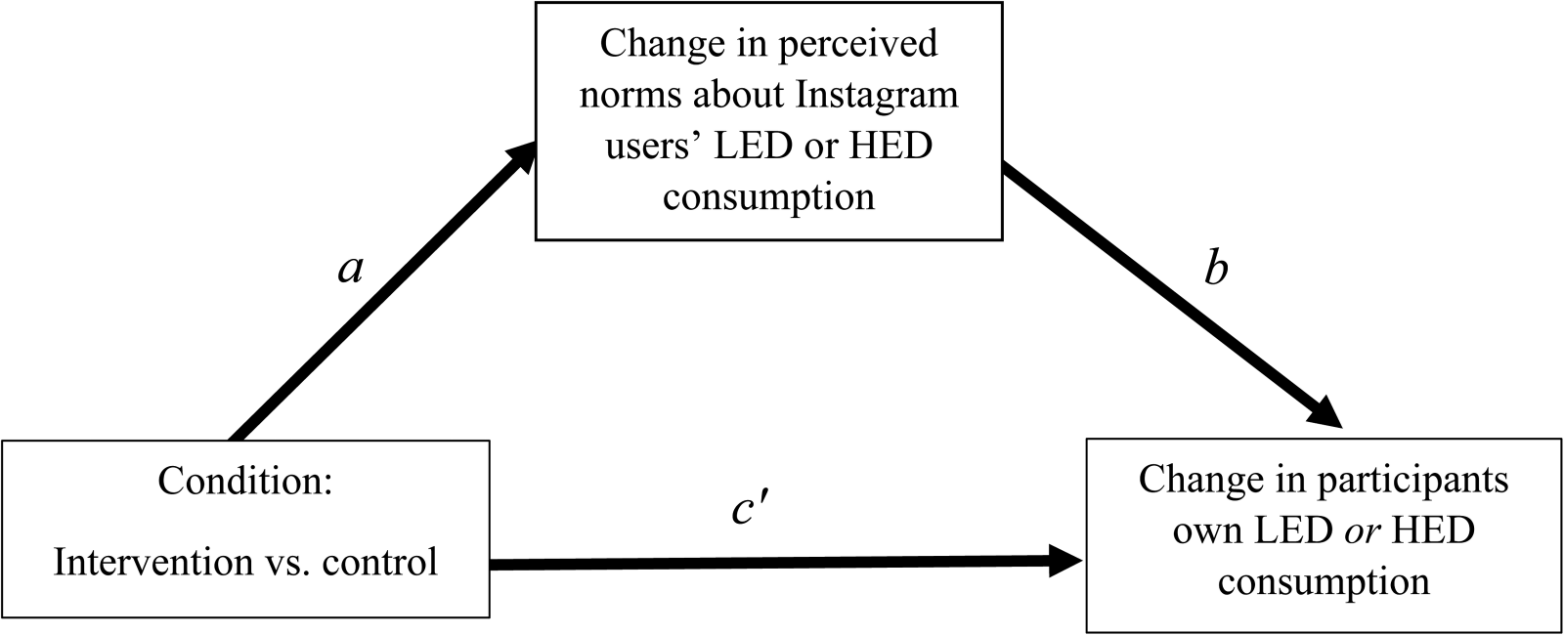
Mediation model of effect of intervention on the change in participants LED consumption via perceived norms about Instagram users’ consumption of LED/HED food across the two-week period. LED: low energy-dense; HED: high energy-dense.

For both LED and HED consumption, there were no significant indirect effects of intervention (versus control) via normative perceptions (i.e., descriptive, injunctive, liking and frequency norms) on participants’ LED or HED consumption (all *p*s > 0.05) (Figure 2).

### Exploratory analysis

Given that there were significant differences between conditions for affiliation with Instagram users, and this has previously been found to be important within social norm effects,^25^ exploratory mediation analysis (Bootstrapping at 5000 samples) was carried out to investigate if these were significant mediators of the effect of the intervention on self-reported consumption. There was a significant indirect effect of the intervention on change in LED foods consumed via identification with Instagram users, *ab* = 0.29, BCa CI [0.11; 0.59], whereby for those in the intervention (vs. control) condition, the more participants identified with Instagram users, the more LED food they consumed. Identification explained around a third of the variance, P_M_ = .28. For change in HED consumption, there was also a significant negative indirect effect of the intervention on change in HED food consumption via identification with Instagram users, *ab* = −0.27 BCa CI [−0.81; −0.02], whereby for those in the intervention (vs. control) condition, the more participants identified with Instagram users, the less HED food they consumed. Identification with Instagram users accounted for around half of the variance, P_M_ = .44.

### Discussion

This study aimed to experimentally test whether following ‘healthy eating’ Instagram accounts compared to interior design accounts, affected participants’ perceptions of what others consume, as well as their own intentions and their self-reported consumption of LED and HED foods. As hypothesised, over two weeks, those in the intervention condition who followed the ‘healthy eating’ accounts significantly increased their self-reported consumption of LED foods (vs. control). Interestingly, they also consumed fewer items of HED foods. However, there were no significant effects of intervention (vs. control) for normative perceptions or intentions to consume LED or HED foods. Further, contrary to predictions, mediation analyses showed that changes in normative perceptions did not mediate the effect of the intervention on self-reported consumption. However, exploratory mediation analyses revealed that measures of affiliation (identification with Instagram users) significantly mediated the effects of the intervention on increased LED consumption, and also, for decreased HED consumption.

Following the healthy eating accounts resulted in a large increase in reported fruit and vegetable consumption, over the two-week period. This is a substantial improvement to previous educational and social media–based interventions also trying to nudge healthier consumption.^16,26^ While exposure to the healthy eating accounts did not test a specific norm directly, in that posts may have conveyed a number of norms that may have been interpreted differently by different participants, this fits with previous research suggesting that norms about others’ fruit and vegetable consumption encourage participants’ consumption of these foods.^7,14,20,21^ The change in self-reported HED consumption is particularly interesting, as although it was not the primary target here (increasing LED consumption was), a reduction in HED consumption coupled with the increase in LED foods, represents a particularly powerful dual route to improving dietary nutrition.

Surprisingly, mediation analyses showed that change scores for perceived descriptive, injunctive, liking and frequency norms, did not mediate the effect of the intervention on participants’ LED consumption or HED consumption. This suggests that the effect of the intervention was not via a shift in perceptions about what others eat, like or approve of. Superficially, this seems at odd with the TPB, and with our previous work, where we show that normative perceptions predict consumption.^21^ However, these null findings may have occurred for a number of different reasons. For instance, the intervention did not significantly shift perceptions; this may be due to the referent group used, or even due to the manipulation deployed here (i.e., our manipulation did not overtly and explicitly convey specific social norms, which may explain the lack of shift in perception). Relatedly, it may be that these perceptions are not influenced as easily, and thus, may not change as readily in response to an intervention or may change more slowly, and thus, the two-week time period was insufficient to detect this. Examining the use of more explicit social norm manipulations, over longer periods of time, would help us to test this.

Putting normative perceptions to one side, the exploratory mediation analyses suggest that other factors, such as affiliation with Instagram users instead may have had an effect on food consumption. Indeed, wanting to be affiliated with and liked by a referent group also leads to following the norm.^27,28^ Here, we observed that affiliation mediated the effect of the intervention, such that feeling more affiliated with users, amplified the effect of the intervention. This demonstrates that identification with the referent group could be an important mechanism through which norms on social media may be followed. It also raises the possibility that interventions that directly target and prime such social media affiliations, may be more important than trying to shift normative perceptions (i.e., it may be that more implicit manipulations coupled with affiliation priming, is preferential to using more explicit manipulations, to shift normative perceptions, in the absence of affiliation priming), though this also requires further investigation.

Notably, there was also no statistically significant effect of the healthy accounts on intentions to consume LED or HED foods. This lack of a significant effect is contrary to the TPB which suggests that intentions are highly important in predicting actual behaviour,^17^ and with previous health interventions utilising the TPB that have successfully increased intentions to consume fruit and vegetables.^19^ Instead, the present study suggests that we perceive others’ behaviour as influential and this may have a stronger influence on actual behaviour than participants’ own intentions or how they think they will be perceived by others (subjective norms). Social norms may therefore be more be useful predictors of actual behaviour, than intentions, as stated by the TPB and subjective norms. However, further work is needed to see if these results can be replicated and are reliable.

### Limitations and future work

This study provides initial evidence that social media could be a useful tool for encouraging positive eating behaviour by young adults. While the long-term effects are not known, those in the intervention condition increased their consumption by 1.37 servings per day over just two weeks, by following the healthy eating accounts. However, there are a number of limitations. The first is that self-report measures were used, and while these are an established and validated method of measuring food consumption (e.g., Refs.^8,24^), they are subject to inaccuracies and social desirability bias in reporting. Thus, it would be useful to objectively examine consumption to confirm that these effects translate to actual eating behaviour (e.g., via measurement of intake in the laboratory). Second, as this was a pilot study, the sample in this study was small, and while large effect sizes were detected, it would be useful to confirm that these effects can be replicated with a larger, community sample. Thirdly, although these results demonstrate an effect at two weeks, further data are required to clarify whether this intervention can produce a long-term change in behaviour that is sustained. Fourth, the study was conducted during the first COVID-19 lockdown and so it is possible that this may have been a confounding factor for participants’ habitual eating behaviour. We also acknowledge that there was no measure of interaction with posts or whether further healthy eating accounts were followed, which may have contributed to intervention effects, and so further studies may benefit from measuring these. While our mediation analyses at least in part suggests that the effects here were driven by affiliation with Instagram users, a further study may also wish to consider the extent to which the effects seen are driven by social influences or whether this is also due to mere exposure to the accounts, as this cannot be ruled out in the current study. Finally, from a manipulation of 5% additional accounts followed, we produced a reasonable increase in LED food consumption in low consumers of fruit and vegetables, and a reasonable decrease in items of HED food items consumed, also. However, it is possible that a stronger manipulation (e.g., 10% additional accounts) could produce an even greater behavioural change, and this is worth exploring, to better establish the parameters of the effect size that can be achieved.

## Conclusions

This pilot study demonstrated that a novel social media–based intervention asking participants to follow healthy eating accounts on the social media site Instagram over two weeks, resulted in an increase in self-reported fruit and vegetable consumption and a decrease in energy dense snacks and sugar sweetened beverages. However, there was no effect on normative perceptions, or intentions, and the effect of the intervention was not mediated by a shift in participants’ perceptions about what they believed Instagram users eat, should eat and like to eat, but by affiliation with the referent group. Overall, this study does provide initial evidence that social media could be a useful tool with which to encourage healthy eating by young adults, in a simple, and cost-effective manner. Further research is now required to examine if these results can be replicated in a larger, more diverse sample.
